# Correlation of fasting blood glucose and haemoglobin A_1c_ measured with an automated analyser

**DOI:** 10.1155/S1463924690000037

**Published:** 1990

**Authors:** Akira Furota, Teikichi Miyagawa, Izumi Tsuda, Noriyuki Tatsumi

**Affiliations:** 1 Department of Clinical and Laboratory Medicine Osaka City University Medical School Asahimachi 1-5-7 Abeno-ku Osaka 545 Japan; 2 Midori Health Care Foundation Tarumicho 3-22 Suita 565 Japan

## Abstract

A subtype of glycohaemoglobin, haemoglobin (Hb) A_1c_, in specimens of whole blood was assayed on a new automated analyser that makes use of high-pressure liquid chromatography. The analyser provided precise and reproducible values. The mean of the HbA_1c_ values was lower than that with an older instrument. The mean tended to increase with the age of the subjects, who were undergoing routine health examinations. No sex difference was found. When measurement was made 1 h after the subjects drank 50g of glucose, the value of HbA_1c_ was unaffected. Correlation was strong between the HbA_1c_ value and the fasting blood glucose value, which suggested that fasting blood glucose could be estimated from the HbA_1c_ value.


Journal of Automatic Chemistry Vol. 12, No. 1 (January–February 1990), pp. 17–21

### Correlation of fasting blood glucose and haemoglobin $A_{1c}$ measured with an automated analyser

**Akira Furota,† Teikichi Miyagawa, Izumi Tsuda,‡ and Noriyuki Tatsumi‡**

*Midori Health Care Foundation, Tarumicho 3-22, Suita 565, and ‡ Department of Clinical and Laboratory Medicine, Osaka City University Medical School, Asahimachi 1-5-7, Abeno-ku, Osaka 545, Japan*

*A subtype of glycohaemoglobin, haemoglobin (Hb) $A_{1c}$, in specimens of whole blood was assayed on a new automated analyser that makes use of high-pressure liquid chromatography. The analyser provided precise and reproducible values. The mean of the $HbA_{1c}$ values was lower than that with an older instrument. The mean tended to increase with the age of the subjects, who were undergoing routine health examinations. No sex difference was found. When measurement was made 1 h after the subjects drank 50 g of glucose, the value of $HbA_{1c}$ was unaffected. Correlation was strong between the $HbA_{1c}$ value and the fasting blood glucose value, which suggested that fasting blood glucose could be estimated from the $HbA_{1c}$ value.*

### Introduction

There are several methods for the measurement of haemoglobin $A_{1c}$ ($HbA_{1c}$) in red blood cells, involving electrophoresis, colorimetry, minicolumns and high-pressure liquid chromatography (HPLC). Each method has different advantages but a problem shared by all of these is the long testing time.

Here, we evaluate the performance of an automated apparatus for the assay of $HbA_1$ and $HbA_{1c}$ (Hi-Auto $A_{1c}$, model 8121, Kyoto Daiichi Kagaku Co., Ltd) first marketed in 1987. In this model, $HbA_{1a}$, $HbA_{1b}$, $HbA_{1c}$, and HbF are separated more completely than in the earlier analyser (model 8120) tested here for comparison, because of changes made in the column filler and in the eluent. In the newer model, unstable $HbA_{1c}$ is eluted first by use of tetrapolyphosphoric acid, which competes for the site on the haemoglobin molecule where glucose binds.

*Table 1. Effects of anticoagulants on $HbA_{1c}$ and $A_1$ values (%) found with models 8120 and 8121.*

| Anticoagulant | Model | $HbA_{1c}$, % | $HbA_1$, % |
|---|---|---|---|
| NaF | 8120 | 5·41 ± 0·51 | 8·09 ± 0·76 |
| (*N* = 204) | 8121 | 4·92 ± 0·44 | 7·09 ± 0·76 |
| EDTA | 8120 | 5·17 ± 0·55 | 8·05 ± 0·81 |
| (*N* = 246) | 8121 | 4·85 ± 0·55 | 6·97 ± 0·84 |
| Na-citrate | 8120 | 5·08 ± 0·36 | 8·07 ± 0·65 |
| (*N* = 85) | 8121 | 4·78 ± 0·38 | 6·80 ± 0·57 |

*Note*: Values for each haemoglobin are the mean percentage ± 1 SD of total haemoglobin, found by HPLC.

† Correspondence to Akira Furota at: Department of Clinical and Laboratory Medicine, Osaka City University Medical School, Asahimachi 1-5-7, Abeno-ku, Osaka 545, Japan

### Materials and methods

### Instruments

The machine tested was a Hi-Auto $A_{1c}$ model 8121, which assays $HbA_{1c}$, $HbA_1$, and HbF in 4 min with 3 μl specimens [1]. For comparison, an older model from the same manufacturer (Hi-Auto $A_{1c}$ model 8120, which resembles the model 8110 described in [2]) was used.

### Materials

Everyone who comes to our health care centre is a company employee with no known current health problems who is to undergo routine health examinations.

*Table 2. Day-to-day reproducibility of $HbA_{1c}$ and $HbA_1$ and retention time in assay of control material. The same column was used to measure about 90 specimens daily for 20 days. Each specimen was from a different subject. Control material was assayed once at the end of each work day.*

| | Item | | | |
|---|---|---|---|---|
| Day | $HbA_{1c}$, % | $HbA_1$, % | Retention time | Numbers of specimens |
| 1 | 6·5 | 9·5 | 2′09 | 90 |
| 2 | 6·4 | 9·3 | 2′08 | 88 |
| 3 | 6·5 | 9·5 | 2′06 | 89 |
| 4 | 6·4 | 9·3 | 2′06 | 90 |
| 5 | 6·5 | 9·5 | 2′11 | 88 |
| 6 | 6·4 | 9·4 | 2′09 | 89 |
| 7 | 6·5 | 9·5 | 2′09 | 91 |
| 8 | 6·4 | 9·3 | 2′11 | 90 |
| 9 | 6·5 | 9·8 | 2′09 | 87 |
| 10 | 6·4 | 9·5 | 2′06 | 90 |
| 11 | 6·5 | 9·5 | 2′09 | 88 |
| 12 | 6·4 | 9·6 | 2′09 | 89 |
| 13 | 6·5 | 9·6 | 2′09 | 89 |
| 14 | 6·5 | 9·5 | 2′09 | 88 |
| 15 | 6·4 | 9·5 | 2′08 | 89 |
| 16 | 6·5 | 9·5 | 2′06 | 92 |
| 17 | 6·4 | 9·5 | 2′09 | 90 |
| 18 | 6·5 | 9·5 | 2′09 | 90 |
| 19 | 6·4 | 9·5 | 2′12 | 89 |
| 20 | 6·5 | 9·5 | 2′06 | 90 |
| CV | 0·79 | 1·18 | 1·37 | Total, 1786 |

0142–0453/90 $3.00 © 1990 Taylor & Francis Ltd.

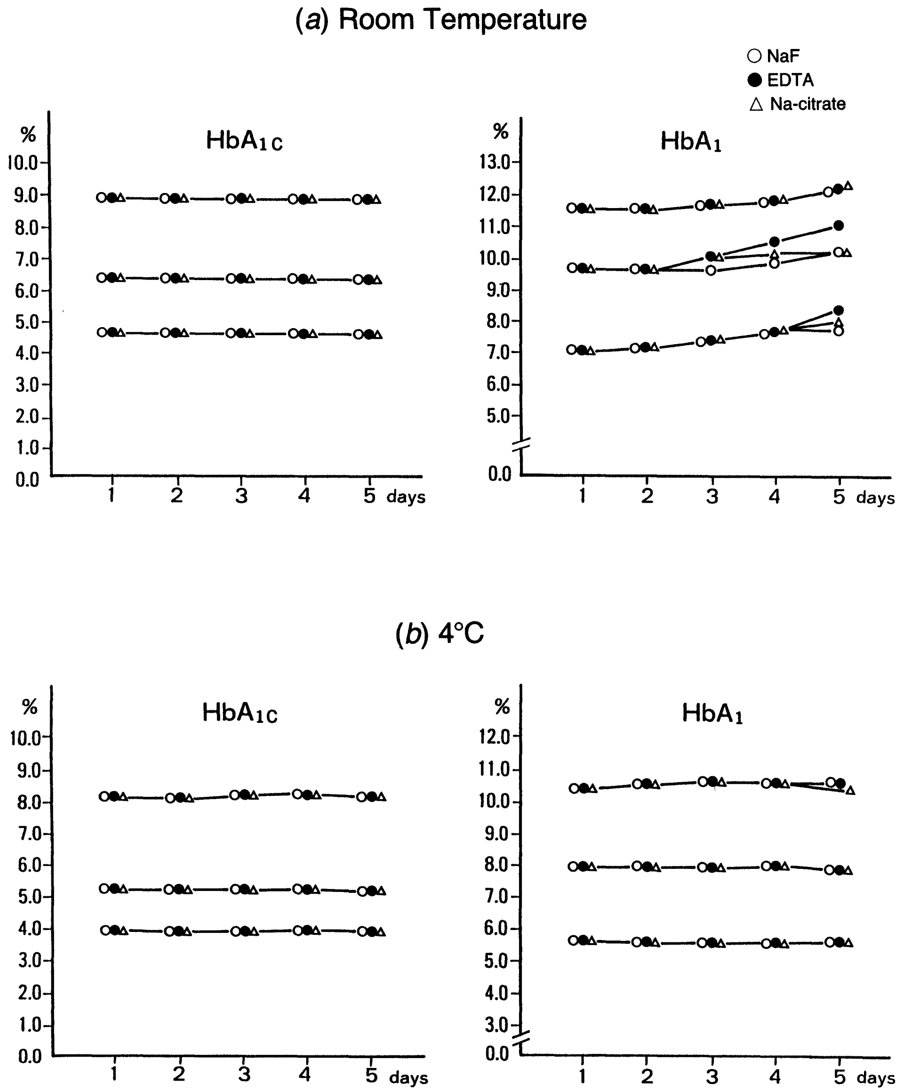

*Figure 1. Effects of storage at two temperatures in three different anticoagulants on values found for $HbA_{1c}$ and $HbA_1$ with the newer analyser. (a) Room temperature; (b) 4 °C. ○, NaF; ●, EDTA; △, Na-citrate.*

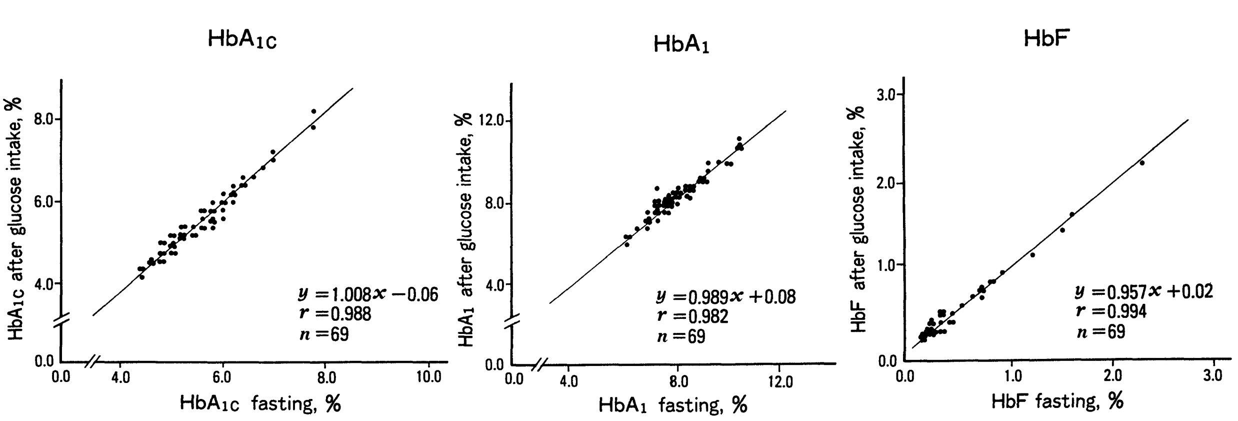

*Figure 2. Comparison of $HbA_{1c}$, $HbA_1$, and HbF values measured with the newer analyser in blood of fasting subjects and in blood of the same subjects one hour after glucose intake.*

Tests of urine, stools, blood chemistry and pulmonary function are carried out, and a complete blood cell count, X-ray films of the chest and the upper gastrointestinal tract, and electrocardiographs are taken. Glucose metabolism is examined by tests of urinary glucose and fasting blood glucose (FBG), and by a 50 g oral glucose tolerance test (OGTT), from which the blood glucose concentration 1 h after the intake of glucose (postprandial blood glucose, or PBG) was used here. In the OGTT, blood was collected into bottles containing NaF when the subject was fasting and again at 1 h.

Venous blood was withdrawn from the examinees with a Venoject syringe (Terumo Co., Osaka) containing NaF (1·25 mg/ml blood), sodium citrate (0·5 ml of a 3·85% solution per 4·5 ml of blood), or a mixture of EDTA-2Na (3·7 mg/ml blood) and heparin (12·5 U/ml blood). The glycohaemoglobins in the blood were assayed within 1 h of sampling. Blood glucose was assayed by the neocuproine method [3].

Control material was purchased from International Reagents Corporation (Kobe).

*Statistics*

Results were evaluated by Student's *t*-test and analysis of variance.

### Results

*$HbA_{1c}$ measurements by the new model*

The effects of the anticoagulant used on the value of $HbA_{1c}$ are shown in table 1. The mean values obtained for $HbA_{1c}$ were highest with NaF, intermediate with EDTA plus heparin, and lowest with sodium citrate. Mean values for $HbA_1$ were almost the same with all three anticoagulants. The newer model gave lower values than the older model in assays of both of these haemoglobins. With NaF, the mean values for $HbA_{1c}$ for the two glycohaemoglobins were significantly different. The differences probably arose from differences in the column fillers and in the elution method, which cause differences in the efficiency of the removal of unstable glycohaemoglobin. With NaF, the blood glucose value of the specimens was not lowered as much as with the other anticoagulants, so that nearly all of the unstable form remained unbound in the specimens. For these reasons, the $HbA_{1c}$ values obtained by the older model, in which removal of the unstable glycohaemoglobin was less efficient, were higher.

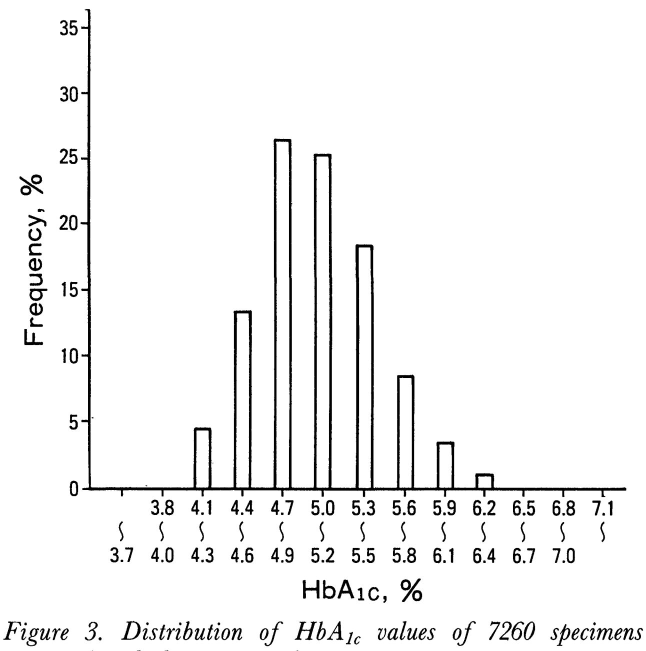

*Figure 3. Distribution of $HbA_{1c}$ values of 7260 specimens measured with the newer analyser.*

When samples were stored at 4 °C or room temperature in one of the three anticoagulants for up to 5 days (figure 1), results for $HbA_{1c}$ stayed about the same. However, at room temperature, values for $HbA_1$ increased during storage.

Table 2 shows the day-to-day reproducibility of $HbA_{1c}$ and $HbA_1$ in control material assayed by the newer model with use of the same column for 20 days. The number of other specimens measured daily was about 90, each from a different subject; in all, 1786 specimens were assayed during these 20 days. Retention time with the HPLC column did not change. Each day, after all test specimens were assayed, control material was tested. Correlation was satisfactory for both glycohaemoglobins. Thus, repeated use such as is described here did not affect the results obtained with the column.

*Relationship betweeen glycohaemoglobin values and blood glucose levels*

In diabetic subjects, the unstable form of $HbA_{1c}$ increases as the glucose level increases but the stable form is unchanged [4]; the same changes occur in healthy subjects, as in the OGTT. Thus, total $HbA_{1c}$ (stable plus unstable forms) rises with increasing glucose. In the 69 subjects chosen at random during the test period to illustrate typical results here, the mean FBG level was 106 ± 17 mg/dl and the PBG level was 161 ± 45 mg/dl. Figure 2 shows the values for each of the three forms of haemoglobin assayed by the new model, $HbA_1$, stable $HbA_{1c}$, and HbF, when the subjects were fasting (*x*-axis) and at 1 h after glucose intake (*y*-axis). Correlation was satisfactory for all these three forms.

*Distribution of $HbA_{1c}$ values in specimens*

Figure 3 shows the distribution of $HbA_{1c}$ values in 7260 examinees screened over a 4 month period. The coefficient of variation (CV) was 5·22% and the standard deviation (SD) was 0·70%. The reference interval was calculated by the method of Hoffman [5]. The mean reference value and the SD were 5·13% and 0·43%, respectively.

Table 3 shows mean values of $HbA_{1c}$ measured with the older model for different age groups and by sex for 5319 subjects. Table 4 shows mean levels of $HbA_{1c}$ in the same way, measured with the newer model for 6807 other subjects. Results in table 4 are based on those in figure 4,

*Table 3. $HbA_{1c}$ values (%) obtained with the older model with subjects classified by age and sex.*

| Sex | Age | | | | | | | Total number |
|---|---|---|---|---|---|---|---|---|
| | −29 | 30− | 40− | 50− | 60− | 70− | Mean | |
| Male | 5·04 (0·26)† | 5·18 (0·35) | 5·31 (0·39) | 5·40 (0·43) | 5·49 (0·46) | 5·52 (0·41) | 5·32 (0·42) | 3660 |
| Female | 4·88 (0·22) | 4·94 (0·26) | 5·02 (0·28) | 5·24 (0·35) | 5·27 (0·32) | 5·24 (0·17) | 5·08 (0·34) | 1659 |
| Total | 4·97 (0·26) | 5·10 (0·35) | 5·22 (0·39) | 5·35 (0·42) | 5·43 (0·44) | 5·45 (0·39) | 5·25 (0·42) | 5319 |

† Numbers in parentheses represent 1 SD.

*Table 4. $HbA_{1c}$ values (%) obtained with the newer model with subjects classified by age and sex.*

| Sex | Age | | | | | | | Total number |
|---|---|---|---|---|---|---|---|---|
| | −29 | 30− | 40− | 50− | 60− | 70− | Mean | |
| Male | 4·93 (0·36)† | 5·07 (0·36) | 5·15 (0·42) | 5·24 (0·45) | 5·30 (0·45) | 5·33 (0·45) | 5·18 (0·44) | 4714 |
| Female | 4·78 (0·30) | 4·91 (0·33) | 4·95 (0·33) | 5·15 (0·37) | 5·25 (0·41) | 5·34 (0·45) | 5·03 (0·38) | 2093 |
| Total | 4·87 (0·36) | 5·02 (0·36) | 5·09 (0·41) | 5·21 (0·43) | 5·29 (0·44) | 5·33 (0·47) | 5·13 (0·43) | 6807 |

† Numbers in parentheses represent 1 SD.

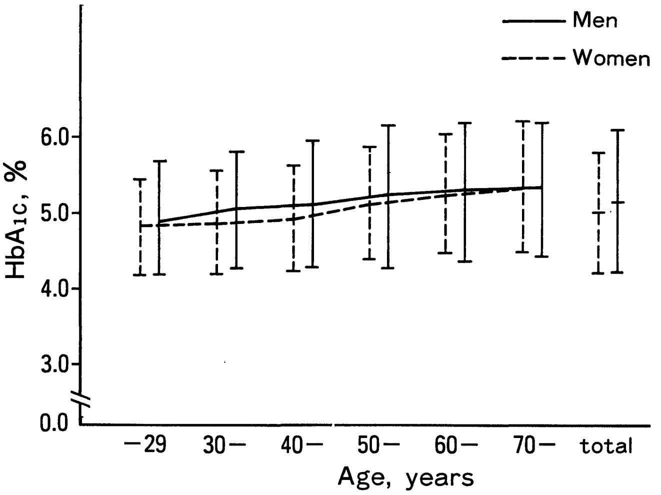

*Figure 4. $HbA_{1c}$ values by age and sex. The values are based on those in table 4, measured with the newer analyser. Curves connect the means and bars show 1 SD.*

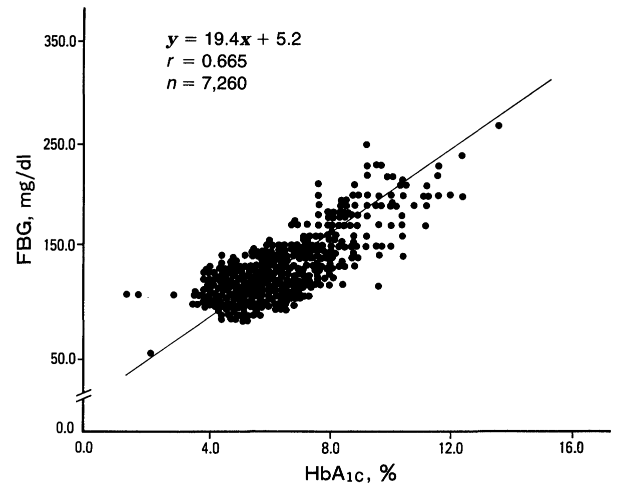

*Figure 5. Correlation between $HbA_{1c}$ and fasting blood glucose (FBG) measured with the newer analyser.*

and one SD is shown. The mean values for this one large group measured with the older model were generally higher than those of the other large group measured with the newer model. $HbA_{1c}$ tended to be higher in men than in women. It also tended to increase with age.

Figure 5 shows the correlation between $HbA_{1c}$ and FBG in the 7260 specimens of figure 3. The correlation was high.

### Discussion

The clinical significance of glycohaemoglobins has been established and their assay is becoming routine. However, the presence of HbF and the instability of $HbA_{1c}$ [4] interfere with the accurate measurement of stable $HbA_{1c}$, which is of clinical interest. The number of samples sent for testing to clinical laboratories is on the increase and automation by a more reliable method is desirable. The machine we tested separates subfractions of haemoglobins in an HPLC column at a controlled temperature, so that measurement is stable and data are both precise and accurate. The machine removed unstable $HbA_{1c}$ and could assay 3 μl samples. The time taken for a single assay was 4 min; the retention time was short (table 2). The newer model gave lower values of $HbA_{1c}$ than the older model, probably because of the removal of unstable $HbA_{1c}$ by the use of tetrapolyphosphoric acid. After blood is sampled, the glucose level decreases and so the unstable $HbA_{1c}$ in the blood changes gradually to the nonglycosylated HbA form [4]. If unstable $HbA_{1c}$ is not removed before assay, then the extent of decomposition will influence the value obtained for total $HbA_{1c}$.

With the newer machine tested here, storage time did not affect the value obtained for $HbA_{1c}$, which value is closely correlated with the presence of diabetes. However, $HbA_1$, which has less correlation with diabetes, was affected by storage time and, if an accurate value for $HbA_1$ is needed, specimens should be assayed within 2 days of sampling. Because $HbA_1$ is unstable when blood is stored, and because correlation is good between $HbA_1$ and $HbA_{1c}$ levels in blood assayed soon after being sampled, in general, assay of $HbA_{1c}$ is more likely to give reliable results. Results for the assay of control material were highly reproducible (table 2).

The anticoagulants did not affect the level of $HbA_1$ and $HbA_{1c}$, so any of them can be used when $HbA_{1c}$ is to be assayed. If the glucose level of the specimen is also to be measured, NaF can and should be used.

The mean difference in the glucose level when 69 of the subjects were fasting and 1 h after the intake of 50 g of glucose was about 50 mg/dl but the levels of $HbA_1$ and $HbA_{1c}$ were unaffected by this difference.

The reference values for $HbA_1$ and $HbA_{1c}$ obtained by groups using cation-exchange chromatography [6] are wider than ours, probably because of the removal of unstable $HbA_{1c}$ by the machine we used. We found that $HbA_{1c}$ tended to increase with the age of our subjects (aged 22 to 82 years). The same tendency has been reported for subjects divided into those aged 21 to their 45th birthday and those older. Because both the blood glucose level and glucose intolerance increase with age [8], the glycohaemoglobin level can be expected to increase, so our findings are reasonable. $HbA_{1c}$ is of use in the monitoring of the glucose levels of a subject in the preceding few months. Because correlation between $HbA_{1c}$ and FBG is high, the value of $HbA_{1c}$ should also be of use for prediction of the likeliness that a subject will develop diabetes mellitus.

### References

1. Nakashima, K., Hattori, Y., Yamazaki, K., Takechi, M., Andoh, Y. and Miyaji, T., *Diabetes*, **39** (1990) (in press).
2. Diamandis, E. P., Daneman, D. and Allen, L. C., *Clinical Chemistry*, **30** (1984), 503.
3. Technicon Instruments Corporation, Technicon Method No. SE4-0002F F4: Technicon AutoAnalyzer II (Technicon Instruments Corp., Tarrytown, New York, 1974).
4. Kawahara, R., Amemiya, T., Komori, T. and Hirata, Y., *Diabetes Care*, **8** (1985), 375.
5. Hoffman, R. G., *Journal of the American Medical Association*, **185** (1963), 864.
6. Paulsen, E. P. and Koury, M., *Diabetes*, **25** (Suppl. 2), (1976), 890.
7. Davis, J. E., McDonald, J. M. and Jarett, L., *Dibaetes*, **27** (1977), 102.
8. Harvey, A. M., Johns, R. J., McKusick, V. A., Owens, A. H., Jr. and Ross, R. S. (Eds), *Practice of Medicine*, 20th edition, (Appleton-Century-Crofts, New York, 1980), p. 798.

